# Wavelet-based detection of transcriptional activity on a novel *Staphylococcus aureus* tiling microarray

**DOI:** 10.1186/1471-2105-13-222

**Published:** 2012-09-05

**Authors:** Víctor Segura, Alejandro Toledo-Arana, Maite Uzqueda, Iñigo Lasa, Arrate Muñoz-Barrutia

**Affiliations:** 1 Genomics, Proteomics and Bioinformatics Unit, Center for Applied Medical Research, University of Navarra, Pamplona, Spain; 2 Laboratory of Microbial Biofilms, Instituto de Agrobiotecnología, Universidad Pública de Navarra-Consejo Superior de Investigaciones Científicas-Gobierno de Navarra, Pamplona 31006, Spain; 3 Cancer Imaging Laboratory, Center for Applied Medical Research, University of Navarra, Pamplona, Spain

## Abstract

**Background:**

High-density oligonucleotide microarray is an appropriate technology for genomic analysis, and is particulary useful in the generation of transcriptional maps, ChIP-on-chip studies and re-sequencing of the genome.Transcriptome analysis of tiling microarray data facilitates the discovery of novel transcripts and the assessment of differential expression in diverse experimental conditions. Although new technologies such as next-generation sequencing have appeared, microarrays might still be useful for the study of small genomes or for the analysis of genomic regions with custom microarrays due to their lower price and good accuracy in expression quantification.

**Results:**

Here, we propose a novel wavelet-based method, named ZCL (zero-crossing lines), for the combined denoising and segmentation of tiling signals. The denoising is performed with the classical SUREshrink method and the detection of transcriptionally active regions is based on the computation of the Continuous Wavelet Transform (CWT). In particular, the detection of the transitions is implemented as the thresholding of the zero-crossing lines. The algorithm described has been applied to the public *Saccharomyces cerevisiae* dataset and it has been compared with two well-known algorithms: pseudo-median sliding window (PMSW) and the structural change model (SCM). As a proof-of-principle, we applied the ZCL algorithm to the analysis of the custom tiling microarray hybridization results of a *S. aureus* mutant deficient in the sigma B transcription factor. The challenge was to identify those transcripts whose expression decreases in the absence of sigma B.

**Conclusions:**

The proposed method archives the best performance in terms of positive predictive value (PPV) while its sensitivity is similar to the other algorithms used for the comparison. The computation time needed to process the transcriptional signals is low as compared with model-based methods and in the same range to those based on the use of filters. Automatic parameter selection has been incorporated and moreover, it can be easily adapted to a parallel implementation. We can conclude that the proposed method is well suited for the analysis of tiling signals, in which transcriptional activity is often hidden in the noise. Finally, the quantification and differential expression analysis of *S. aureus* dataset have demonstrated the valuable utility of this novel device to the biological analysis of the *S. aureus* transcriptome.

## Background

The complete deciphering of the information contained in the genome would be helpful to improve our understanding of the biological processes occurring in living organisms. High-density oligonucleotide-based whole-genome microarray is an extensively used technology to detect the expression of all RNA species including protein coding RNAs and non-coding RNAs. It is particularly suitable for the analysis of whole small-sized genomes such as those corresponding to bacteria. For these organisms high resolution can be achieved with the microarrays currently provided by the manufactures.

Applications of tiling array technology include the generation of transcriptional maps and annotations of genomes, the identification of transcription factor binding sites, the analysis of alternative splicing events, the analysis of methylation states, the discovery of genotyping and polymorphism, and the re-sequentation of genomes [1].

The emerging high-throughput next generation DNA sequencing (NGS) technologies [2] have revolutionized transcriptomics by allowing RNA analysis through cDNA sequencing on a massive scale (RNA-seq). Several limitations inherent to microarray technologies are overcome by NGS technologies, in particular, it is not necessary to design appropriate probes and the experimental reproducibility is guaranteed. However, the microarray design presented in [3] allowed a comprehensive examination of gene expression and genome-wide identification of alternative splicing as well as detection of coding and noncoding transcripts. This microarray (Affymetrix GG-H array) was compared with RNA-seq in [3]. The reproducibility in the estimation of gene and exon abundance was high and even more sensitive than RNA-seq at the exon level. This microarray design contains as targets near 50000 highly transcribed fragments of unknown functions from Affymetrix tiling microarray data [4]. The NGS experiments highlighted that 49% of these fragments had uniquely mapped reads, revealing a high degree of concordance between both technologies.

The analysis of a tiling microarray experiment starts with a two-step process that generates a discrete signal. First, the DNA or RNA samples are hybridized in the custom designed tiling array. Second, for each probe, the raw intensities are converted to a score [5]. The result is a discrete intensity signal with a value per probe.

**Figure 1:**
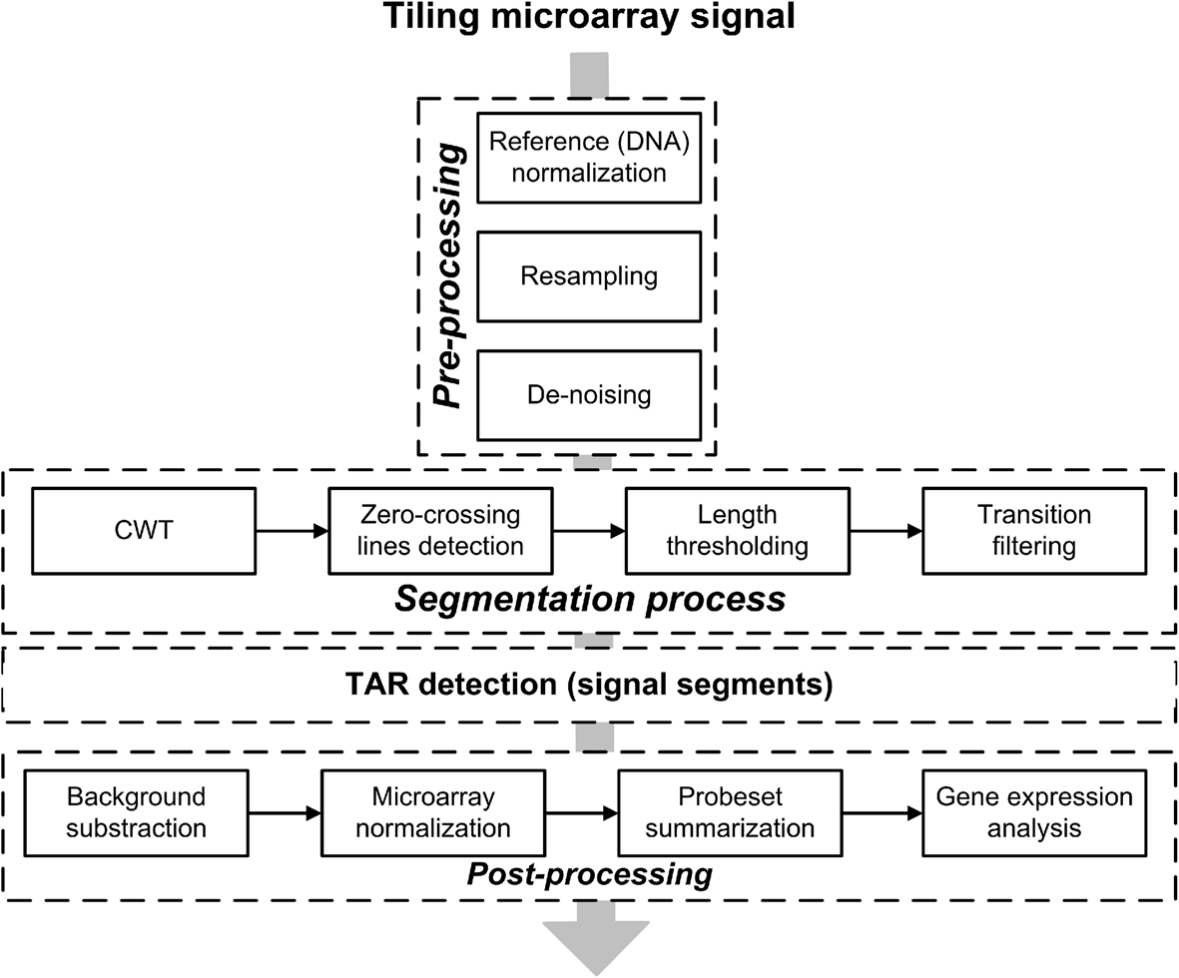
**Wavelet-based processing of tiling signals.** Workflow for the analysis of the tiling signal based on the computation of the Continuous Wavelet Transform (CWT).

The workflow shown in Figure 1 summarizes the methodology. It consists of four basic blocks: (1) signal pre-processing (DNA normalization, non-uniform to uniform resampling and de-noising); (2) segmentation to detect abrupt intensity changes; (3) definition of transcriptionally active regions (TARs) and (4) biological knowledge extraction (for example, differential expression analysis of genes).

Transcriptome analysis refers to the detection of segments where the noisy tiling signal is constant. The start and end points of these segments correspond to transcript start and end sites. Several approaches have been deployed in the segmentation of tiling signals: pseudo-median or Hodges-Lehmann estimator [6,7], local non parametric smoothing [8,9], hidden Markov models [10-13], circular binary segmentation [14] and structural change model [15,16].

Wavelet analysis using the Discrete Wavelet Transform (DWT) [17] has demonstrated excellent performance in the analysis of ChIP-chip experiments using tiling array technology [18,19]. In this paper, we propose a wavelet transform based method for the identification of TARs in tiling signals (ZCL). We have chosen the SUREShrink algorithm for denoising and a method based on the computation of the Continuous Wavelet Transform (CWT) for detection of transcription start and end sites. In particular, the sharp transitions of the tiling signal are identified as the zero-crossing lines of a multiresolution decomposition using as the mother wavelet the second derivative of a Gaussian [20]. We applied the proposed analysis to the public *Saccharomyces Cerevisiae* dataset to validate our analytical approach. The novel identification algorithm was compared with two well-known methods: pseudo-median sliding window (PMSW) and structural change model (SCM). The absence of a biologically validated ground truth to evaluate the resulting segmentations prevent the use of specificity and sensitivity as performance metrics. Consequently, the evaluation has been made in terms of positive predictive value (PPV), sensitivity and computation time. We also evaluated the segmentation quality resulting from the combination of the TARs detected by several of the methods under study.

We also used this algorithm for the identification of the subset of transcripts whose expression decreases in a *S. aureus* strain deficient in the sigma B transcription factor. SigB has been shown to be involved in the stress response to different stimuli, the regulation of *sarA*, *sarH1*, and *agr* that control a wide array of virulence factors, biofilm formation, the ability of *S. aureus* to bind to various host-cell matrix proteins such as fibrinogen and fibronectin, and in the development of resistance to the antibiotics methicillin and teicoplanin [21-26].

We applied the segmentation methods to this high quality dataset and we have demonstrated its usefulness for the analysis of the tiling array derived transcriptome map. The results demonstrate that ZCL not only allows a rapid identification of the transcripst based on the segmentation procedure but also a more accurate estimation of the expression level of each transcript.

## Results and discussion

All the steps needed to obtain a trancriptional map from the raw data (read the CEL files, normalize, denoise and segment the tiling signal) have been implemented using the statistical language *R/Bioconductor*[27]. The CRAN packages *Rwave*, *wavethresh* and *wmtsa* have been used for wavelet analysis. All the R functions described are available as Supplementary Material (Additional file 1, Additional file 2 and Additional file 3). The R code to perform the example analyses and the generation of the figures included in the paper can also be found as Supplementary Material. The results show that wavelets compare well with the rest of methods in terms of segmentation accuracy and time consumed in the analysis.

### Experimental datasets

#### Saccharomyces cerevisiae dataset

The dataset is described in [16]. An oligonucleotide array for *S. cerevisiae* was developed. It contains 6.5 million probes and interrogates both strands of the full genomic sequence with 25-mer probes tiled at an average of 8 nucleotide intervals on each strand and 4 nucleotide tile offset between strands. The first-strand cDNA was synthesized using random primers from poly(A) and total RNA. A set of genomic DNA was also hybridized for normalization purposes [15]. Their analysis of the transcription map identified the transcript boundaries, its structure and the intensity level of coding and non-coding transcripts [16]. All data (CEL files, bmap files for both strands and annotation file) was deposited in ArrayExpress database with accession number E-TABM-14.

#### Staphylococcus aureus dataset

The *Staphylococcus aureus* custom tiling microarray (NA-Staph-b520729F) was designed in collaboration with Affymetrix (Santa Clara, CA, USA). Specifically, the microarray (format 49-7875 with 11 *μ*m features) contains a total of 522,406 probes, divided into two parts. The first part corresponds to the tiling array containing a total of 384,932 probes (25-mer), which are further divided into eight sets. The set used in our analysis covers both strands of the *S. aureus* NCTC 8325 genome (2,821,347 bp covered by 363,127 probes). Each 25-mer probe was tiled each 14-nt across the whole genome, resulting in 11-nt overlaps and a 7-nt tile offset between strands. The microarray design has been deposited in the ArrayExpress Archive at EMBL-EBI (http://www.ebi.ac.uk/microarray-as/ae/), ArrayExpress accesion no. A-AFFY-165.

Before cDNA synthesis, RNA integrity from each sample was confirmed on Agilent RNA Nano LabChips (Agilent Technologies). 10 *μ*g of RNAs extracted from bacterial strains grown until exponential phase (OD600nm = 0.8) were reverse transcribed using SuperScript II reverse transcriptase (Invitrogen Life Technologies). They were processed following the protocol of the Affymetrix GeneChip Expression Analysis Technical Manual (P/N 702232 Rev. 2) in the presence of 6 ng/ml Actinomycin D to avoid spurious second-strand cDNA synthesis during the reverse transcription reaction [28]. Sense RNA corresponding to B. subtilis poly-A *lys*, *phe*, *thr*, *trp*, *dap* genes were spiked into sample RNA as a control for the labeling and hybridization steps. cDNA was digested by DNase I (PIERCE) in 10X DNAse I buffer (USB-Affymetrix) and the size of digestion products was analyzed in the Agilent Bioanalyser 2100 using RNA Nano LabChips to ensure that the fragmentation resulted in a majority of products in the range of 50 to 200 base-pairs. The fragmented cDNA were then biotinylated using terminal deoxynucleotidyl transferase (Promega) and the GeneChip DNA labeling reagent (Affymetrix) following the manufacturer’s recommendations. Biotinylated cDNA (5 *μ*g per array) were hybridized for 16 hours according to the Affymetrix protocol in a total volume of 200 *μ*l per hybridization chamber. Following incubation, the arrays were washed and stained in the Fluidics station 450 (Affymetrix) using the protocol FS450_0005. The arrays were then scanned using the GeneChip scanner 3000 (Affymetrix). The intensity signals of each probe cell were computed by the GeneChip operating software (GCOS) and stored in cell intensity files (.CEL extension) before preprocessing and analysis. All microarray data described in this study have been deposited in the ArrayExpress Archive at EMBL-EBI (http://www.ebi.ac.uk/microarray-as/ae/), ArrayExpress accesion no. E-MEXP-2778.

### Probe annotation and normalization

The annotation of the PM probe sequences was obtained with the alignment to the genome sequence of *S. cerevisiae* strain S288c (SGD of August 7, 2005) as provided in the package *davidTiling* of Bioconductor. Available data correspond to 3 replicates of poly(A), 2 replicates of total RNA and 3 replicates of genomic DNA. The CEL files were read and the normalized signals (poly(A) and total RNA) were obtained using Equation 4. The analysis steps (denoising, segmentation and detection of TARs) were performed on the poly(A) signal as it showed an improved hybridization quality [16]. Once the signal is constructed from CEL and annotation files we used *tilingArray* package functions to obtain equally-spaced samples. Other resampling methods can be applied without loss of generality.

The annotation files for *S. aureus* microarray are provided in the ArrayExpress database (A-AFFY-165). The microarrays of the experiment correspond to three replicates of genomic DNA, three replicates of RNA of the 15981 wild-type strain, and three replicates of the *sigB* deletion. All the preprocessing steps were performed as previosly described for *S. cerevisiae* dataset.

### Denoising

**Figure 2:**
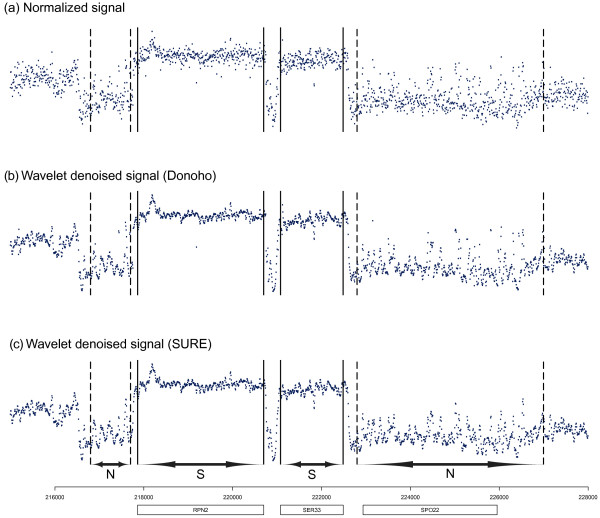
**Signal to noise ratio of different filtering methods.** Portion of the tiling signal used to evaluate the Signal to Noise Ratio (SNR). We consider two signal regions (S) and two noise regions (N). **(a)** Normalized signal. **(b)** Denoised signal using Donoho’s threshold. **(c)** Denoised signal using the SUREShrink threshold.

The denoising was evaluated using the signal to noise ratio (SNR), a quantitative measure of its performance. In order to compare the results obtained with those from Huber et al. [15] based on a variance stabilization and normalization transformation, the same definition of SNR was used. We looked at a set of control regions, two positive control regions (pos) within the ORFs of RPN2 and SER33 at coordinates 217860−220697 and 221078−222487and two negative control regions (neg) in the background at coordinates 216800−217700 and 222800−227000 of *S. cerevisiae* (see Figure 2). We assumed (as in [15]) that the differences between positive and negative controls give an estimation of the signal level, whereas variations from the mean intensity within each region are due to noise. 

The SNR was computed as

(1) $$\text{SNR}=\frac{\Delta \mu }{\sigma }=\frac{1}{\sigma }\left(\underset{r\in \text{pos}}{\sum }\frac{\mu_{r}}{\left|\text{pos}\right|}-\underset{r\in \text{neg}}{\sum }\frac{\mu_{r}}{\left|\text{neg}\right|}\right),$$

with the noise standard deviation *σ*calculated as the average of the differences between 0.975 and 0.025 quantiles of the data within each of the control regions. Namely,

(2) $$\sigma =\frac{\underset{r\in \text{pos},\text{neg}}{\sum }(Q_{r}^{0.975}-Q_{r}^{0.025})}{\left(Q_{N}^{0.975}-Q_{N}^{0.025})(|\text{pos}|+|\text{neg}|\right)}$$

where the symbol *r* counts over the different regions and *Q*_*N*_ refers to the standard normal distribution N(0,1). Table 1 shows the SNR of the normalized signal and the wavelet-based denoised signal using Donoho’s method [29] and the SUREShrink approach [30] in relation to the best SNR obtained in [15]. We observe that the use of wavelets for denoising results in a large increase in the SNR (18.94*%* with Donoho’s method and 30.63*%*with SUREShrink approach), especially when the SUREShrink denoising is applied. This could be due to the elimination of most part of the non-Gaussian noise component and the consequent reduction in the estimated variance. In the rest of the paper, the SUREShrink is the method applied for denoising. 

**Table 1:** **Estimated SNR values of the tiling signal shown in Figure**3

**SNR results**	
Signal	SNR
Best SNR in [15]	4.58
Normalized signal	4.28
Wavelet denoising (Donoho’s)	5.28
Wavelet denoising (SUREShrink)	**6.17**

**Figure 3:**
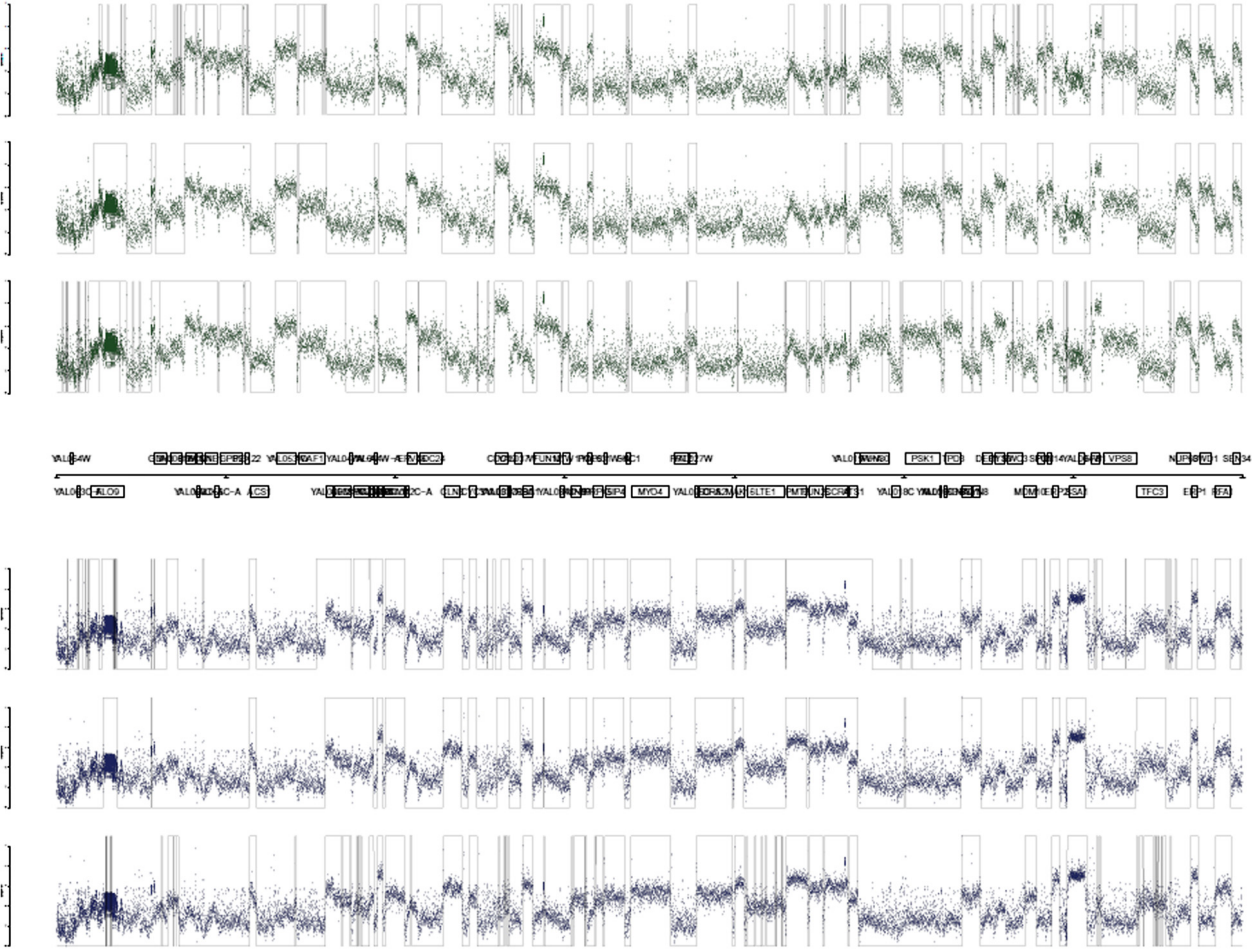
**Wavelet-based segmentation of *S. cerevisiae *tiling signal.** Visualization of *S. cerevisiae* tiling microarray signal along 140 kb of chromosome 1. Each dot corresponds to a probe of the forward strand (top) and reverse strand (bottom). The superimposed pulse signal represents the segmentation obtained using the different methods (PMSW, SCM, ZCL). The parameters of the different analysis are described in the Results and discussion section of the manuscript.

Estimated SNR values of the tiling signal. The normalized and the wavelet denoised signal using Donoho’s and SUREShrink on which the calculation was performed are shown in Figure 3.

### Segmentation

A descriptive example of the denoising and segmentation for *S. cerevisiae* is shown in Figure 3. The analysis corresponds to a 140 Kb segment of chromosome 1 from position 20000 to position 160000. The results are given for the three algorithms compared (SCM, PMSW, ZCL). The CWT computation of ZCL used as mother wavelet the second derivative of a Gaussian with 100 scales. Zero-crossing lines were calculated and only those with a length greater than a pre-defined threshold were considered to correspond to signal transitions.

The TAR start and end positions were defined as the transition locations for which the difference between the mean intensity of neighboring segments is greater than 10% of the dynamic range of the tiling signal. Moreover, the inspection of the intensity histogram of chromosome 1 forward strand was used to set the minimum normalized transcription level value to −2. The same parameters were adopted to process the other strands of the organism. The R function *segmentZCL* (provided as Supplementary Material) implements the whole segmentation procedure.

**Figure 4:**
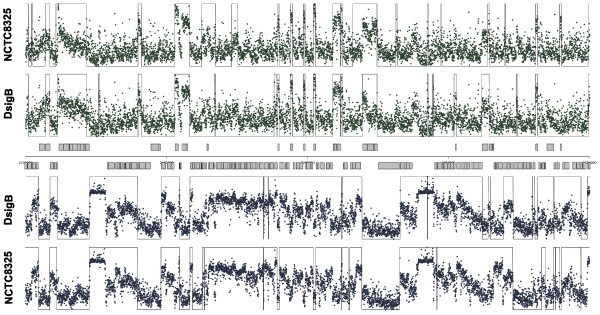
**Wavelet-based segmentation of *S. aureus *tiling signal.** Visualization of *S. aureus* tiling microarray signal along 200 kb of NCTC8325 genome. Each dot corresponds to a probe of the forward strand (top) and reverse strand (bottom) for *sigB* mutant and 15981 wild-type normalized signals. The superimposed pulse signal represents the segmentation obtained using ZCL. The parameters of the analysis are described in the Results and discussion section of the manuscript.

Another representative example of segmentation results is given in Figure 4. In this case, the *S. aureus* signal of 15981 and *sigB* mutant were segmented using ZCL and fixing only the number of scales to 100. No denoising was applied prior to the segmentation. The figure represent a fragment of the signals from position 2.1 Mb to 2.3 Mb. In Additional file 4: Figure S1 and Additional file 5: Figure S2, the results for the PMSW algorithm are shown. In Additional file 6: Figure S3 and Additional file 7: Figure S4, the results for the SCM algorithm are presented.

### Segmentations comparison using *S. cerevisiae* dataset

The results from the ZCL segmentation were compared to those obtained with PSMW [6] and SCM [15]. The robust PMSW method is based on the calculation of a pseudo-median within a sliding window. The local expression level is computed with the Hodges-Lehmann estimator [31] on the RNA normalized signal. To be able to do this, the *Tilescope* pipeline [7] was implemented. Once the candidate transcript regions were determined, the TARs were assembled by the combination of a normalization intensity threshold and a max-gap and min-run criteria. The former is defined as the maximum distance below which two adjacent transcribed probes are included in the same TAR. The later as the minimum length of a feature to be classified as a transcribed region.

Huber’s method is based on the structural change model (SCM). The SCM model [15,16] is used in econometrics for the modeling of sharp transitions in financial time series. It has been applied to the segmentation of comparative genomics hybridization (CGH) data [32]. The signal is modeled as a piecewise constant function of chromosomal coordinates described using the segment boundaries, the maximum number of segments and the mean signal value for each segment. The method is applied independently to each chromosome and, if the signal is strand-specific, to each of its two strands. A dynamic programming algorithm part of the *tilingArray* package of Bioconductor computes a globally optimal set of parameters for segmentations of increasing number of segments.

Due to the lack of a biologically validated ground truth to evaluate the outputs, we compared the methods in terms of two metrics, sensitivity and positive predictive value (PPV) at probe-level. We define sensitivity as the number of probes in the detected TARs that overlap with annotated regions (true positives, *TP*) divided by the total number of probes in the annotated regions (sum of true positives and false negatives, *TP* + *FN*): Sensitivity=*TP*/(*TP* + *FN*). The PPV is defined as the number of probes in the detected TARs that overlap with annotated regions (*TP*) divided by the total number of probes in the detected TARs (sum of true and false positives, *TP* + *FP*): *PPV*=*TP*/(*TP* + *FP*). A sensitivity of 100% is not expected since in any given tissue or cell line at any given experimental condition, not all known genes will be expressed. Also, a PPV of 100% is not expected since an accurate and complete gene annotation is not available [5,33].

The PMSW and SCM methods were applied to the *S. cerevisiae* using the parameters previously reported in the literature [6,15]. In particular, the PMSW method used a bandwidth size (BW) of 3, a normalized intensity threshold equal to −2, a separation between the probes in a TAR (maxgap) of 10 and a minimum acceptable TAR size (minrun) of 90. The maximum number of segments for the SCM method was fixed to 1500. In the case of ZCL we selected the following parameters: 100 wavelet scales, minimum TAR size of 10 and minimum value of normalized intensity transcription equal to −2. The zero-crossing line length threshold was computed based on the histogram of line lengths.

**Figure 5:**
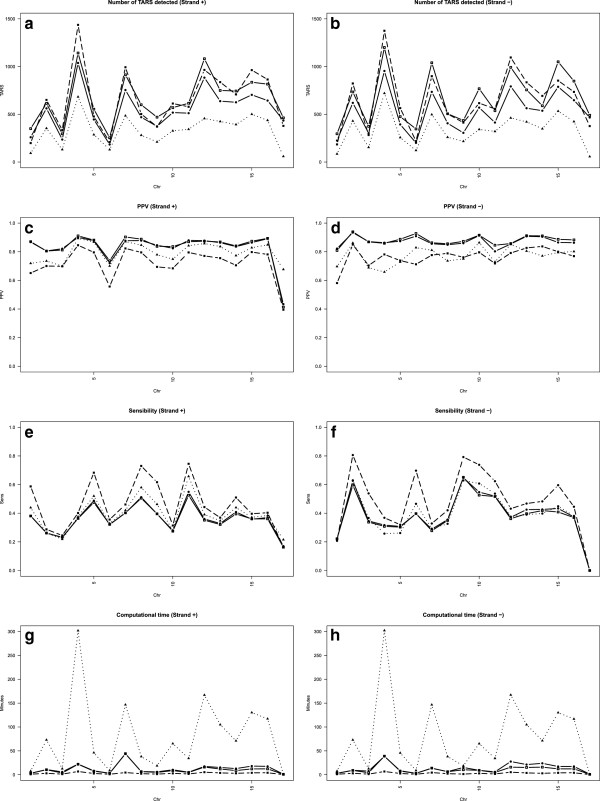
**Results for the identification of TARs.** Number of detected TARs, probe-level PPV and sensitivity, and computation time for the proposed (solid line), PMSW (dashed line) and SCM (dotted line) methods. The analysis was performed for the forward (left) and the reverse (right) strands of all chromosomes of *S. cerevisiae* tiling microarray data.

The graphical representation of the results obtained after processing the *S. cerevisiae* tiling signal are shown in Figure 5. For each chromosome we calculated the number of detected TARs, PPV, sensitivity and computation time for the forward and reverse strands. Table 2 presents the performance metrics mean value. The segmentation with PMSW includes a larger number of detected TARs. The highest PPV values are obtained with the ZCL method (with or without denoising) at the cost of a slightly reduced sensitivity. PMSW and ZCL outperform SCM in term of computation time. The best sensitivity value corresponds to PMSW.

**Table 2:** **Evaluation metrics for *S. cerevisiae *dataset**

**Evaluation metrics (*S. cerevisiae*)**				
**Method**	**TARs**	**PPV**	**Sensitivity**	**Time (min)**
PMSW	22114	0.7416	**0.4700**	2.88
SCM	11246	0.7847	0.3904	79.09
ZCL	18209	0.8486	0.3760	13.02
ZCLSure	22513	**0.8547**	0.3686	10.70

Mean number of detected TARs, probe-level PPV, probe-level sensitivity and computational time for PMSW, SCM and ZCL methods (all chomosome and strands of *S. cerevisiae*).

In-depth analysis of chromosome 1 gives interesting insights into concerning the relationship between methods. In the forward strand, the number of probes annotated as genes is 12796 representing 19.35% of the total number of probes. 65.16% of probes are correctly classified by the three algorithms (12.21% of gene probes and 52.95% of non-gene probes). From the annotated probes, 63.12% are detected by all methods, while only 11.85% of the probes are not detected by either of them. This means that 88.15% of the annotated probes are detected by at least one of the methods. The reverse strand contains 11866 annotated probes (17.90% of probes located in this strand), from which 19.74% are considered part of a TAR by all the methods and 28.35% are true negative probes. In this strand, 51.41% of the annotated probes are included in a TAR by any method while only 14.35% are never detected. In other words, 85.65% of the probes in the strand are detected by at least one algorithm. In light of this outcome, we considered it worthwhile to evaluate if the combination of results computed with the different methods would improve the performance of the segmentation.

#### Combination of TAR probes candidates

We evaluated the improvement in performance obtained by the combination of the different segmentations. We chose different strategies to define the sets (intersection of two or three methods and majority voting system). After a decision is taken on the candidates, TARs are constructed to create the transcriptional map. In Table 3, we give evaluation metrics (PPV and sensitivity) for both strands of *S. cerevisiae* chromosome 1. As individual methods, ZCL gives the best PPV and the best sensitivity for the reverse strand. The best performing combination considering a compromise between PPV and sensitivity is given by the majority voting system.

**Table 3:** Evaluation of segmentation combinations for both strand of chromosome 1

**Integrative transcriptional analysis**				
**Method**	**PPV Forward**	**Sensitivity Forward**	**PPV Reverse**	**Sensitivity Reverse**
PMSW	0.6511	0.5873	0.5811	0.2073
SCM	0.7188	0.4390	0.6968	0.2146
ZCL	**0.8675**	0.3821	**0.8220**	**0.2208**
PMSW ⋂ SCM ⋂ ZCL	0.6312	**0.5984**	0.5441	0.2030
PMSW ⋂ ZCL	0.6448	0.5921	0.5744	0.2076
PMSW ⋂ SCM	0.6370	0.5957	0.5504	0.2043
SCM ⋂ ZCL	0.7053	0.4409	0.6626	0.2116
Majority voting	0.7247	0.4396	0.6993	0.2164

PPV and sensitivity for both strand of chromosome 1 using individual TAR detection algorithms and the combination of their results.

#### Computational performance

*S. cerevisiae* analyses were executed in an Intel(R) Xeon(R) processor server (64 bits, 4 cores, 2 GHz) with 32 Gb installed memory running Red Hat Enterprise Linux AS release 4 and R 2.13.0. Computing times needed to process each chromosome strand with the described methods are shown in Figure 5. For the same signal length, longer computation time is required for SCM, while comparable times are needed for PMSW and ZCL. The mean time to segment the whole transcriptome is 2.88 mins for PMSW, 13.02 mins for ZCL and 79.09 mins for SCM.

### Differential expression analysis of *S. aureus sigmaB* mutant

Comparative segmentation analysis using ZCL and PMSW and SCM algorithms was applied for the hybridization data obtained with a custom designed Affymetrix tiling array of *S. aureus*. Segmentation results for *S. aureus* are summarized in Table 4. In this case, the performance measures are almost identical for all methods. These results suggest that the performance of the methods depends on the quality of the signals, decreasing for PMSW and SCM algorithms as the SNR of the signal get worse. In spite of this, other advantages such as computation time, automatic selection of parameters and the possibility of parallel computation makes ZCL our preferred option to segment tiling signals.

**Table 4:** Evaluation metrics for S. aureus dataset

**Evaluation metrics (S. aureus)**					
**Tiling Signal**	**Metric**	**PMSW**	**SCM**	**ZCL **	**ZCLSure **
WT Forward	PPV	0.6298	**0.6498**	0.6248	0.6407
WT Forward	Sens	0.8657	**0.8766**	0.8715	0.8719
WT Reverse	PPV	0.6867	0.6993	**0.7050**	0.6989
WT Reverse	Sens	0.8506	**0.8560**	0.8388	0.8535
*Δ*sigB Forward	PPV	0.6238	**0.6388**	0.6227	0.6308
*Δ*sigB Forward	Sens	**0.9054**	0.9035	0.9027	0.9036
*Δ*sigB Reverse	PPV	0.6664	**0.6815**	0.6765	0.6748
*Δ*sigB Reverse	Sens	**0.8697**	0.8684	0.8667	0.8515

Mean number of detected TARs, probe-level PPV, probe-level sensitivity and computational time for PMSW, SCM and ZCL methods.

The most frequent transcriptional analysis is the detection of genes that have changed their expression in the conditions under study (differential expression analysis). As sigma B affects the expression of more than one hundred genes, we decided to test whether it is possible to use the intensity of all the probes included in each detected TAR with the ZCL segmentation procedure to calculate the expression level of the transcript in a particular environmental condition. In order to carry out this analysis using tiling microarrays we need to compress the intensity of all the probes included in each detected TAR into one value. Standard methods for microarray normalization can be applied, for example RMA (Robust Multichip Average) algorithm in the case of Affymetrix microarrays [34]. This processing can be performed using the packages *affxparser*, *affy* and *limma* of *Bioconductor* for CDF (chip definition file) generation, normalization and differential expression analysis.

**Figure 6:**
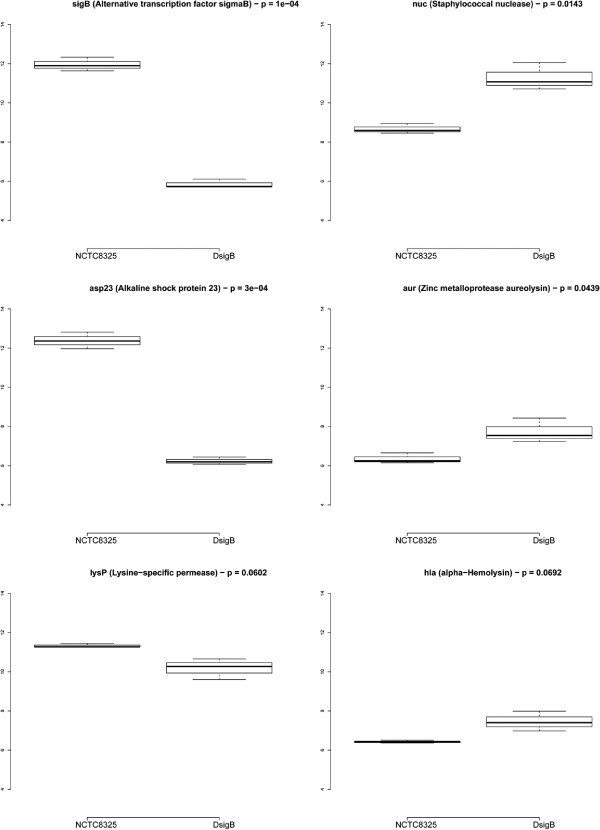
**Differential expression analysis of *S. aureus sigB *mutant.** Boxplots of median gene expression intensities. The expression of the selected genes has been previously reported to change in response to sigB repression.

We introduced a simple analytical tool to be used independently of the microarray platform to measure the gene expression level based on the median value of the TAR probe intensities. We calculated this value for each wild-type and sigmaB mutant sample. We applied a statistical analysis (t-test) to obtained the p-value associated with the expression change taking into account the biological variability of the samples. Considering well-defined TARs in the *S. aureus* annotation, we found previously described alterations in several genes [21]. In Figure 6, we show the boxplots that represents these expression level changes. We confirmed the down-regulation of sigB and other *σ*^*B*^-regulated genes, as the alkaline shock protein 23 (*asp23*) [22,23] and lysine-specific permease (*lysP*) [21], although the latter is not statistically significant (p > 0.05). We also found genes up-regulated in the sigmaB mutant, as the staphylococcal nuclease (*nuc*) [23,24], the zinc metalloprotease aureolysin (*aur*) [24,25] and the *α*-hemolysin (*hla*) [24,26], the latter without a statistically significant p-value. 

## Conclusions

Transcriptomics is a powerful technology for the study of gene structures and RNA-based regulation in any organism. Genome-wide transcriptome analysis of prokaryotes can be carried out with any of these two techniques: RNA-seq and genomic tiling arrays [35]. High-resolution tiling arrays have been used, among others, to study the transcriptomes of *Caulobacter crescentus*[36], *Escherichia coli* and [37], *Listeria monocytogenes*[38].

In this paper, we propose a combined WT-based method for the denoising and segmentation of tiling signals. For illustrative and evaluative purposes, we applied the proposed analysis to the public *S. cerevisiae*. Our denoising results show an increase in the SNR of the filtered signal with respect to Huber’s method [15]. We believe it is advisable to properly denoise the tiling signal before segmentation as the number of false positives induced by signal variability is thus reduced. Even when constructing a manual segmentation, it seems an advisable choice to mark the transitions on the denoised signal, as its improved quality could help the expert to better discriminate between low expression transcripts and noise.

Our segmentation algorithm (ZCL) calculates all the possible break points based on the zero-crossing lines of the second derivative of the Gaussian wavelet. The results show that our method achieves the best compromise between accuracy (evaluated in terms of PPV and sensitivity) and computation time. The R code provided can be used to apply our algorithm as well as to combine the resulting segmentation with other methods as PMSW and SCM.

We also designed a new tiling microarray for the analysis of *S. aureus* genome, publicly available in the ArrayExpress database (accession number A-AFFY-165). This platform has been used for the comparison of the gene expression pattern of the *S. aureus* 15981 wild type and its isogenic *sigB* mutant. We selected this mutant because it is one of the most study staphylococcal regulatory factors and consequenctly it was a useful gold standard to compare the accuracy of our algorithms. The relevance of the segmentation results comes from the fact that a correct analysis of the tiling signals could improve the matching between the probes and the corresponding transcriptional units. In particular, it could help to more precisely localize the start and end transcription sites or even, include units that are not annotated in the current genome definition.

Once the TARs are properly detected, differentially expressed transcripts can be identified by well-known methods (such as Linear Models for Microarray Data (LIMMA) [39]) with a previous probe summarization algorithm to generate the transcript annotation (using, for example, Robust Microarray Analysis (RMA) [34]). In practice, this means that differential gene expression analysis could benefit from an enhanced analysis of tiling signals such as the one proposed here. To confirm the accuracy of the proposed method, we introduced a simple measure based on the median of TAR probe intensity. Using this approach, known up-regulated (*nuc*, *aur*, *hla*) and down-regulated (*asp23*, *lysP*) genes in sigB mutant were verified.

In conclusion, we present a novel method for denoising and segmentation of tiling microarray signals based on wavelet multiresolution analysis that outperforms previous methods in terms of SNR, positive predictive value and computation time. The R code that implements the method is given as supplementary material and can be easily adapted to a parallel computing schema. Also, we have introduced the possibility of combining the results of ZCL with those obtained with other two well-known approaches (PMSW and SCM) for the segmentation of tiling signals.

## Methods

### WT-based analysis

The CWT of a continuous signal *s*(*x*)is defined as [40]

(3) $$\mathrm{CWT}(a,b)=\frac{1}{\sqrt{a}}\overset{+\infty }{\underset{-\infty }{\int }}s(x)\psi^{*}\left(\frac{b-x}{a}\right)\mathrm{dx}$$

where $a\in R^{+}-\{0\}$ is the scale, b∈R is the translation, *ψ*(*x*) is the mother wavelet, *ψ*^∗^((*b*−*x*)/*a*) is the complex conjugated, scaled and translated wavelet and *CWT* is the 2D matrix of wavelet coefficients. The continuous input signal *s*(*x*) interpolates the discrete input samples *sk*,*k*=1,…,*n*where *n* is the length of the signal.

The CWT can be interpreted as the correlation of the input signal with a position reversed version of *ψ* rescaled by a factor *a*. For an 1D input signal, the result is a 2D description of the signal with respect to the position *b* and scale *a* and shifted by *b*. The scale *a* is inversely proportional to the central frequency of the dilated wavelet *ψ*_*a*_=*ψ*(*x*/*a*), which is typically a bandpass function; *b* represents the position location at which we analyze the signal. The larger the scale *a*, the wider the analyzing function *ψ*_*a*_, and hence the smaller the corresponding analyzed frequency. The output value is maximized when the frequency of the signal matches that of the corresponding dilated wavelet. The CWT computation for arbitrary scales can be easily adapted to a parallel implementation with a linear computational complexity [41].

Mallat’s fast wavelet algorithm [42] uses the multiresolution properties of the wavelet to compute the CWT at dyadic scales *a*=2^*i*^ and time shifts *b*=2^*i*^*k*, k∈,Z, resulting in what is known as DWT. For additional information about the wavelet transform and its properties the reader is referred to [17].

### Normalization of tiling microarray data

The analysis starts with background correction and quantile normalization as describe by the RMA algorithm [34]. Next, we calculate the geometric mean of the RNA intensities and the geometric mean of the DNA replicates to get a signal score *sk* at position *k* proportional to the transcription level in the reference genome [16]

(4) $$s[k]=\frac{\overset{n}{\underset{j=1}{\sum }}\log{\text{RNA}}_{j}[k]}{\overset{m}{\underset{j=1}{\sum }}\log{\text{DNA}}_{j}[k]},$$

where *n* is the number of RNA samples and *m* is the number of DNA samples.

### WT-based denoising

One of the most established methods of wavelet-based denoising was proposed by Donoho and Johnstone [29] and it is based on the thresholding of the DWT coefficients at scale *a*=2. This method is composed of three steps: (i) calculate the DWT of the tiling signal *sk* at scale *a*=2; (ii) threshold the wavelet coefficients; (iii) compute the inverse wavelet transform of the thresholded coefficients. A universal threshold, *T*, is proposed [29] to remove white noise which it is given by

(5) $$T=\sigma \sqrt{2\log(n)}\;\;\text{with}\;\;\sigma =\text{MAD}/0.6745,$$

where *n* is the length of *s*, *σ*is the noise level and MAD is the estimated median absolute deviation in the first scale. An important issue is the selection of a suitable wavelet function. As the signal can be roughly approximated to a zero-order polynomial, a boxcar-like function such as the Haar wavelet gives a reasonable level of correlation (i.e., a good pattern matching) with the target signal.

Another well established method of wavelet shrinkage is SUREShrink [30]. This is based on Stein’s Unbiased Estimator for Risk (SURE). A subband adaptive threshold is applied. If the wavelet coefficients in the *jth* subband are {*x*_*i*_:*i*=1,…,*n*}, we consider a soft thresholding procedure and apply Stein’s result. The quantity

(6) $$\text{SURE}(T;x)=n-2\cdot ♯\{i:|x_{i}|\leq T\}+\overset{n}{\underset{i=1}{\sum }}{\left(x_{i}\wedge T\right)}^{2}$$

is an unbiased estimate of risk, where *T* is the threshold and *x*_*i*_∧*t*=min(*x*_*i*_*T*). This estimator can be used to select a threshold:

(7) $$T_{\text{SURE}}=\mathrm{argmi}n_{0\leq T\leq \sqrt{2\mathrm{logn}}}\text{SURE}(T;x)$$

For a large dimension *n* the law of large numbers will ensure that *T*_SURE_ will be almost the optimal threshold [30].

### WT-based segmentation

An important issue in signal processing is to define an appropriate representation able to compress most of the signal information into few representative features. Sharp variations in amplitude (i.e., transitions and peaks) are among the most meaningful features of a signal. For that reason, many segmentation algorithms rely on their detection. Previous studies have detected the peaks in mass spectrometry data using either the ridge lines [43] or the zero-crossing lines [44] in a multi-scale decomposition of the signal. Zero-crossing lines seems a more consistent description as they belong to connected curves, are more robust to noise and easier to detect that ridge lines [44].

It has been previously shown that the position of multiscale sharp transitions can be obtained from the zero-crossings of the signal convolved with the Laplacian of a Gaussian [45]. We define a wavelet at scale *a* as

(8) $$\psi (x)=\frac{d^{2}\theta_{a}(x)}{dx^{2}}$$

where *θ*_*a*_ is a Gaussian function dilated by a factor *a*. Since the wavelet transform can be represented as

(9) $$\mathrm{CWT}(a,b)=(s*\psi_{a})(x){|}_{x=b}$$

we derive that

(10) $$\begin{array}{l} \mathrm{CWT}(a,b)=\left[s*\left(a^{2}\frac{d^{2}\theta_{a}}{dx^{2}}\right)\right](x){|}_{x=b}\\ \;=a^{2}\frac{d^{2}}{dx^{2}}(s*\theta_{a})(x){|}_{x=b} \end{array}$$

Hence, the wavelet transform of *s*(*x*) is proportional to the second derivative of *s*(*x*)smoothed by *θ*_*a*_(*x*). The zero-crossings of *CWT*(*ab*) correspond to the inflection points of *s*∗*θ*_*a*_. The identification of transcript start and end sites is achieved by computation of the redundant CWT over a wide scale range followed by zero-crossing line detection and length thresholding. The chosen mother wavelet is the second derivative of a Gaussian. The redundancy of the CWT yields enhanced information on the position-scale localization of the features of interest (in this case, the transitions) [46].

**Figure 7:**
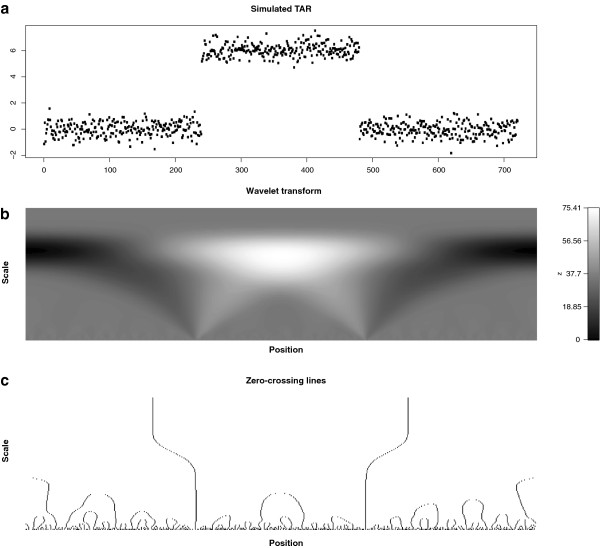
**Zero-crossing lines of the second derivative Gaussian wavelet.** An illustration of zero crossing lines detection. **(a)** Box signal contaminated with additive Gaussian noise (standard deviation = 0.5). **(b)** Absolute values of the CWT coefficients. The second derivative of the Gaussian was used as the mother wavelet. **(c)** All zero-crossing lines are shown. Note how the two longest lines correspond to the two sharp transitions of the box signal.

An illustrative example is given in Figure 7. We generated a simulated transcriptional unit with a rectangular pulse signal of 721 samples with additive Gaussian noise (mean 0 and standard deviation 0.5) (see Figure 7(a)). The absolute values of the wavelet transform coefficients and the zero crossing lines are shown in Figure 7(b) and (c), respectively. Observe how the position of these lines corresponds to abrupt intensity transitions in the noisy signal and the longest connected curves identify the start and end points of the rectangular pulse. The R functions provided as supplementary material detect the zero-crossing lines and identify them as transcription start sites (TSS) and transcription end sites (TES) depending on the slope sign.

### Identification of transcriptional active regions

The candidates start and end sites detected as described in the previous section, are filtered to remove incorrect assignments. The purpose of this procedure is to filter those transitions that do not correspond to variations in signal intensity. For the generation of TARs we considered the signal transitions in which variation in intensity is at least 10% of the dynamic range of the analyzed signal. We also eliminate from the list of detected TAR all the start and end points that are not correctly paired off. We use the sign of the zero-crossing lines to separate start and end points and we match each start site with its corresponding end site. Finally, we define the minimum normalized intensity threshold required for the segments to be considered as transcriptional active regions. This value is calculated as the median of the signal intensity distribution, but this threshold can also be user-defined. In order to improve the definition of TARs, we cluster together consecutive segments for which the mean normalized intensity value is over the threshold.

## Competing interests

The authors declare that they have no competing interests.

## Authors’ contributions

VS and AMB conceived the idea, developed the methods and implemented the software. ATA and IL design the NA-Staph-b520729F microarray and MU and ATA carried out the processing and hybridization of samples. ATA, IL and VS made the biological interpretation of the results. All authors participated in writing and revising the manuscript.

## Supplementary Material

Additional file 1 **R code: Segmentation and visualization functions.** Implemented functions in R language to perform PMSW and SCM segmentation and the proposed wavelet-based method for denoising and segmentation. In addition, functions are provided for proper visualization of data, integration of analysis results and evaluation of the obtained transcriptional maps. Click here for file

Additional file 2 **R code: Segmentation analysis of *S. cerevisiae.*** R script for segmentation of the *S. cerevisiae* dataset and the generation of the figures included in the manuscript. Click here for file

Additional file 3 **R code: Segmentation analysis of *S. aureus*).** R script for the segmentation of the *S. aureus* dataset and the generation of the figures included in the manuscript. Click here for file

Additional file 4 **Figure S1.** Visualization of *S. aureus* tiling microarray signal along 200 kb of NCTC8325 genome. Each dot corresponds to a probe of the forward strand (top) and reverse strand (bottom) for NCTC8325 wild-type normalized signal. Superimposed pulse signal represents the segmentation obtained using PMSW method. The parameters of the analysis are described in the Results and discussion section of the manuscript. Click here for file

Additional file 5 **Figure S2.** Visualization of *S. aureus* tiling microarray signal along 200 kb of NCTC8325 genome. Each dot corresponds to a probe of the forward strand (top) and reverse strand (bottom) for sigmaB mutant normalized signal. Superimposed pulse signal represents the segmentation obtained using PMSW method. The parameters of the analysis are described in the Results and discussion section of the manuscript. Click here for file

Additional file 6 **Figure S3.** Visualization of *S. aureus* tiling microarray signal along 200 kb of NCTC8325 genome. Each dot corresponds to a probe of the forward strand (top) and reverse strand (bottom) for NCTC8325 wild-type normalized signal. Superimposed pulse signal represents the segmentation obtained using SCM method. The parameters of the analysis are described in the Results and discussion section of the manuscript. Click here for file

Additional file 7 **Figure S4.** Visualization of *S. aureus* tiling microarray signal along 200 kb of NCTC8325 genome. Each dot corresponds to a probe of the forward strand (top) and reverse strand (bottom) for sigmaB mutant normalized signal. Superimposed pulse signal represents the segmentation obtained using SCM method. The parameters of the analysis are described in the Results and discussion section of the manuscript. Click here for file
