# Supplementary figures and images for: Wavelet-based detection of transcriptional activity on a novel Staphylococcus aureus tiling microarray
Source: BMC Bioinformatics. 2012 Sep 5;13:222. doi: 10.1186/1471-2105-13-222 (PMC3563573; doi:10.1186/1471-2105-13-222)

expression level

0.0  
-0.5  
-1.0  
-1.5

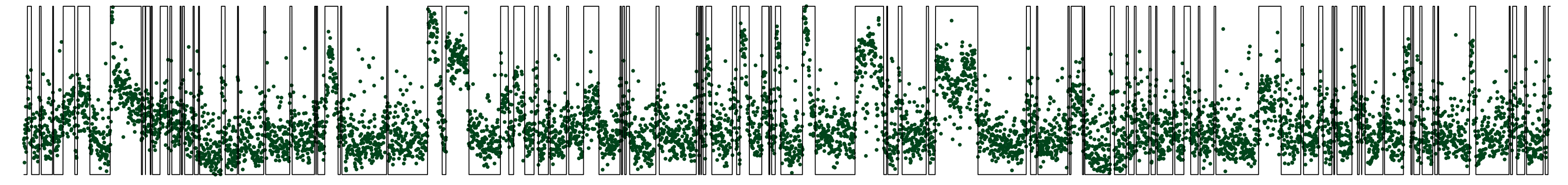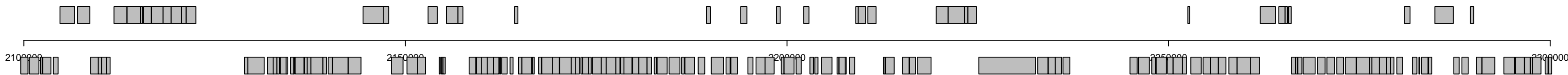

expression level

0.5  
0.0  
-0.5  
-1.0  
-1.5

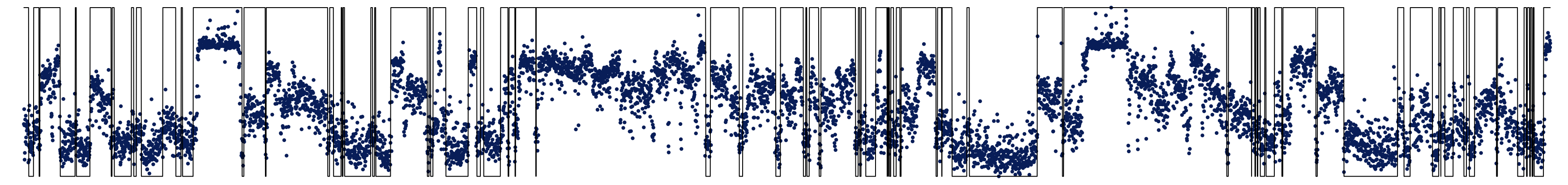

Supplement: Additional file 4 — Figure S1. Visualization of S. aureus tiling microarray signal along 200 kb of NCTC8325 genome. Each dot corresponds to a probe of the forward strand (top) and reverse strand (bottom) for NCTC8325 wild-type normalized signal. Superimposed pulse signal represents the segmentation obtained using PMSW method. The parameters of the analysis are described in the Results and discussion section of the manuscript. [file 1471-2105-13-222-S4.pdf]

expression level

0.0  
-0.5  
-1.0  
-1.5

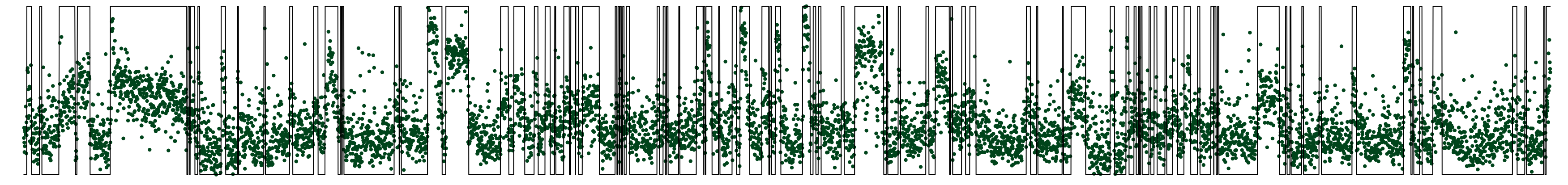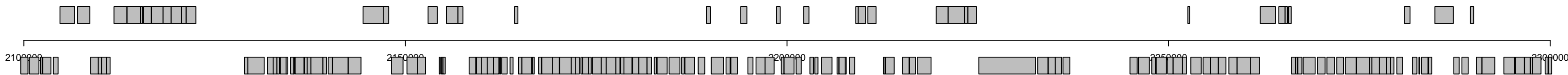

expression level

0.5  
0.0  
-0.5  
-1.0  
-1.5

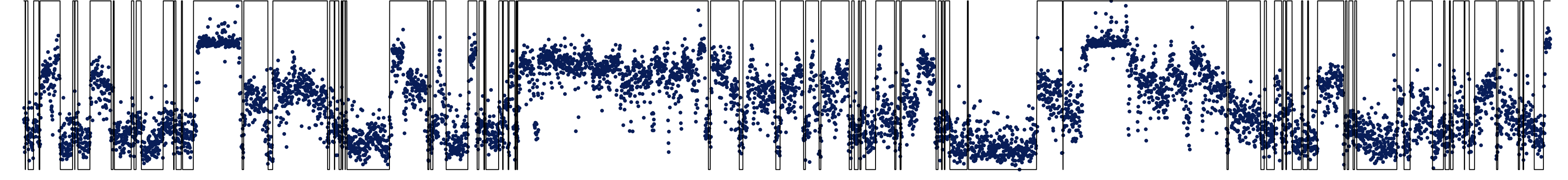

Supplement: Additional file 5 — Figure S2. Visualization of S. aureus tiling microarray signal along 200 kb of NCTC8325 genome. Each dot corresponds to a probe of the forward strand (top) and reverse strand (bottom) for sigmaB mutant normalized signal. Superimposed pulse signal represents the segmentation obtained using PMSW method. The parameters of the analysis are described in the Results and discussion section of the manuscript. [file 1471-2105-13-222-S5.pdf]

expression level

0.0  
-0.5  
-1.0  
-1.5

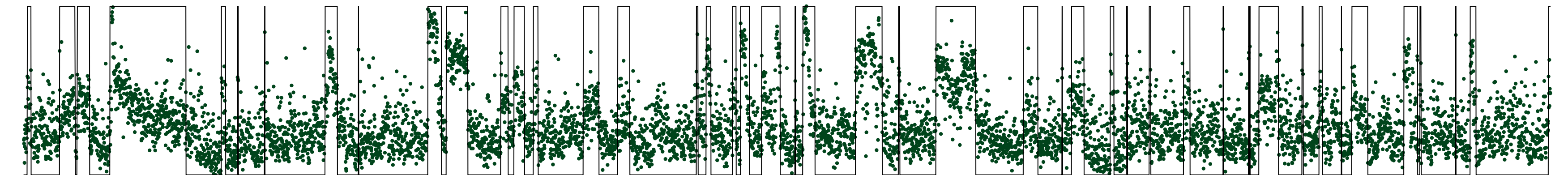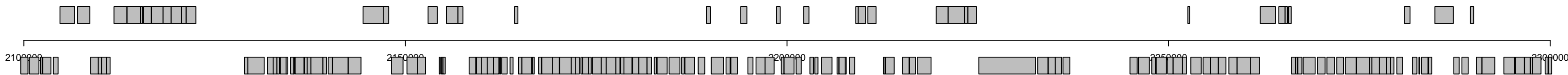

expression level

0.5  
0.0  
-0.5  
-1.0  
-1.5

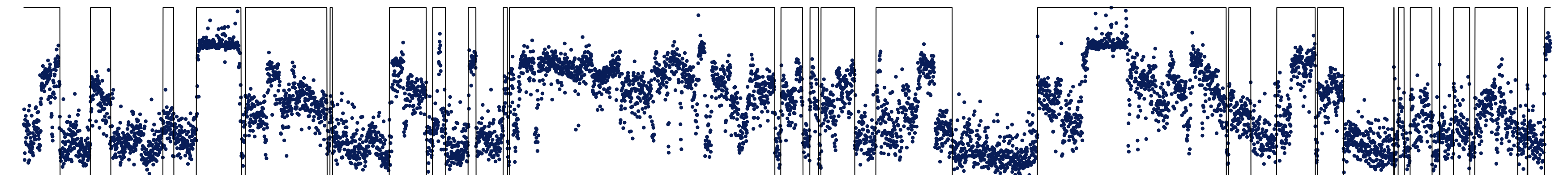

Supplement: Additional file 6 — Figure S3. Visualization of S. aureus tiling microarray signal along 200 kb of NCTC8325 genome. Each dot corresponds to a probe of the forward strand (top) and reverse strand (bottom) for NCTC8325 wild-type normalized signal. Superimposed pulse signal represents the segmentation obtained using SCM method. The parameters of the analysis are described in the Results and discussion section of the manuscript. [file 1471-2105-13-222-S6.pdf]

expression level

0.0  
-0.5  
-1.0  
-1.5

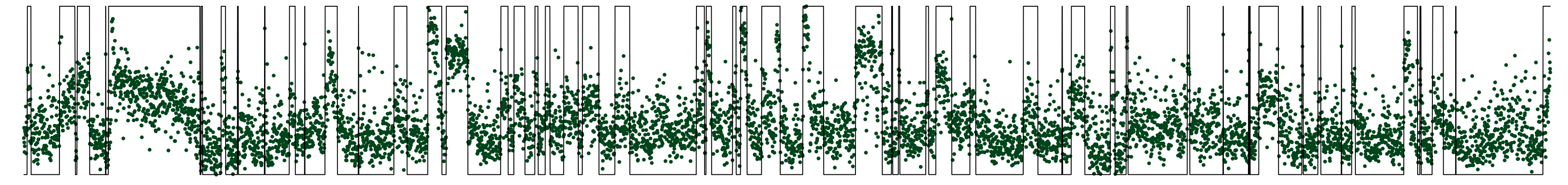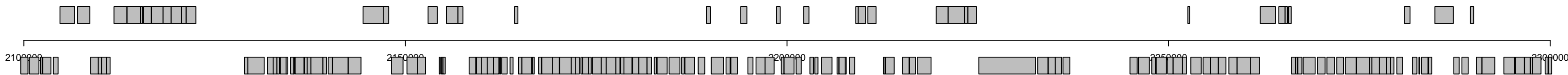

expression level

0.5  
0.0  
-0.5  
-1.0  
-1.5

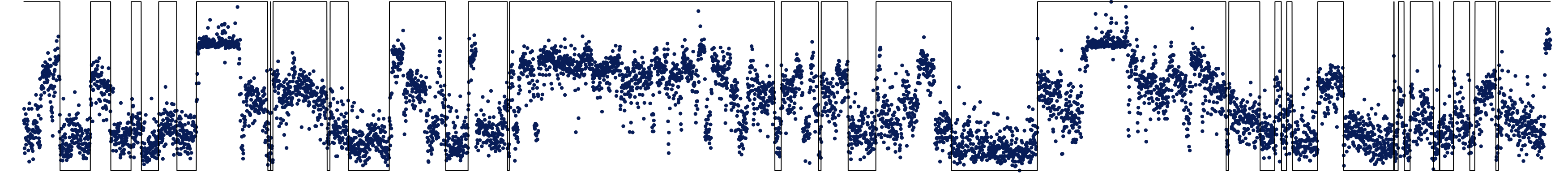

Supplement: Additional file 7 — Figure S4. Visualization of S. aureus tiling microarray signal along 200 kb of NCTC8325 genome. Each dot corresponds to a probe of the forward strand (top) and reverse strand (bottom) for sigmaB mutant normalized signal. Superimposed pulse signal represents the segmentation obtained using SCM method. The parameters of the analysis are described in the Results and discussion section of the manuscript. [file 1471-2105-13-222-S7.pdf]
